# The Transcriptional Regulatory Network of *Mycobacterium tuberculosis*

**DOI:** 10.1371/journal.pone.0022178

**Published:** 2011-07-19

**Authors:** Joaquín Sanz, Jorge Navarro, Ainhoa Arbués, Carlos Martín, Pedro C. Marijuán, Yamir Moreno

**Affiliations:** 1 Instituto de Biocomputación y Física de Sistemas Complejos, Universidad de Zaragoza, Zaragoza, Spain; 2 Departamento de Fsica de Materia Condensada, Universidad de Zaragoza, Zaragoza, Spain; 3 Grupo de Bioinformación, Instituto Aragonés de Ciencias de la Salud, Zaragoza, Spain; 4 Grupo de Genética de Micobacterias, Departamento de Microbiología, Facultad de Medicina, Universidad de Zaragoza, Zaragoza, Spain; 5 Centro de Investigación Biomédica en Red de Enfermedades Respiratorias, Facultad de Medicina, Universidad de Zaragoza, Zaragoza, Spain; 6 Departamento de Física Teórica, Universidad de Zaragoza, Zaragoza, Spain; Hopital Raymond Poincare - Universite Versailles St. Quentin, France

## Abstract

Under the perspectives of network science and systems biology, the characterization of transcriptional regulatory (TR) networks beyond the context of model organisms offers a versatile tool whose potential remains yet mainly unexplored. In this work, we present an updated version of the TR network of *Mycobacterium tuberculosis* (*M.tb*), which incorporates newly characterized transcriptional regulations coming from 31 recent, different experimental works available in the literature. As a result of the incorporation of these data, the new network doubles the size of previous data collections, incorporating more than a third of the entire genome of the bacterium. We also present an exhaustive topological analysis of the new assembled network, focusing on the statistical characterization of motifs significances and the comparison with other model organisms. The expanded *M.tb* transcriptional regulatory network, considering its volume and completeness, constitutes an important resource for diverse tasks such as dynamic modeling of gene expression and signaling processes, computational reliability determination or protein function prediction, being the latter of particular relevance, given that the function of only a small percent of the proteins of *M.tb* is known.

## Introduction

During recent years, simulations of biological systems have been spurred by the massive acquisition and availability of data in molecular and cell biology. It is increasingly becoming evident that simulations can be paired with experiments, and in fact, they are customarily used by computational scientists to understand the quantitative behavior of many complex biological systems. Additionally, *in silico* simulations are also successfully employed in the design of new biomolecular experiments thus driving experimentalists. Although the gap between *in vivo* and *in silico* biology has been remarkably reduced, there are still many limitations hindering the adoption of computational approaches in everyday biomolecular research. Filling in this gap will help for a better understanding of mechanisms and operation of cellular processes.

Achieving such a goal is not easy. On the one hand, experimental data for large biological systems are often incomplete and of non-uniform quality, so that their modeling is often hampered by the lack of complete knowledge of the cellular circuitry or networks of interactions. On the other hand, a successful *in silico* model would require an enormous amount of information relevant to the system under analysis, which posses constraints to the number of variables that can be used to characterize the state of a biological system. Our still limited capability to produce accurate computational models of living systems is however producing simulation tools useful in drawing new principles and laws, both from the topology and the dynamics of the system under consideration, that complement the huge body of experimental work. In particular, viewing the system as a network has revealed as a powerful approach that allows elucidating its components and their dynamical interplay in order to understand the functioning of the system as a whole. By constructing and analyzing biological networks one can overcome the limitations of traditional reductionism reasoning.

In this paper, we study the transcriptional regulatory network of one of today's most threatening menaces: *Mycobacterium tuberculosis*, that causes Tuberculosis (TB). *M.tb* is an extraordinarily successful pathogen that currently infects approximately one-third of the global population and causes 8 million new cases of tuberculosis annually [1]. Along with AIDS and malaria, it remains being one of the deadliest diseases worldwide, with 1.8 millions deaths each year [2]. As a major threat to human health globally, there is an urgent need to improve our knowledge on the molecular and systemic mechanisms underlying the pathological success of this bacillus. Herein, the combination attempted of genomics, bioinformatics, and systems biology, together with network science and topological analysis, may be useful to generate new analytic tools and to suggest further therapeutic strategies.

The success of the pathogen is based on the singularities of its life cycle within the host organism. As was pointed in [3], the characteristic features of the tubercle bacillus include its slow growth, dormancy, complex cell envelope, intracellular pathogenesis and genetic homogeneity. As an intracellular pathogen, the bacillus must be able to gain entry into macrophages, disorganize the phagosome maturation and its fusion with the lisosome, multiply intracellularly, survive within the lung granulomas for years, and disperse to a new host via aerosols [4]. The ability to persist for long periods in the host depends on the capacity of *M.tb* to acquire and utilize nutrients from the very interior of the macrophage phagosome. The bacillus switches metabolic pathways to utilize fatty acids rather than carbohydrates during infection [5]. It is therefore likely that the expression of different sets of genes at various stages of infection is crucial to its survival. For instance, the *phoP* gene regulates a large number of genes of *M.tb* bacilli, controlling many metabolic functions: hypoxia response, respiratory metabolism, response to stress, lipid synthesis, etc. [6].

Unraveling the biochemical mechanisms behind *M.tb* infection is crucial for the development of new drugs and vaccines aimed at eradicating the disease. The current TB vaccine, called BCG (live attenuated strain derived from *Mycobacterium bovis*), remains the most widely used worldwide, but the degree of protection it confers is very variable and rather inadequate against the respiratory form of TB [7], [8]. Consequently, several teams have been working on the development of new vaccines [8], [9]. One of such candidates is a live vaccine that consists of a mutant strain of *M.tb* called SO2, characterized by inactivation of *phoP* gene, which greatly attenuates its virulence. It is therefore of utmost importance to know in depth as many aspects as possible of the biochemical and metabolic networks of *M.tb* through the application of existing analytic tools, including those of systems biology and signaling science [10].

Such a challenging goal would imply to comprehend both the transcriptional control of the signaling system and the interaction of the bacillus with the immune system of the host. As a first step, it is necessary to study the backbone of this complex system, i.e., the circuitry behind the biochemical processes operating at the gene expression and signaling levels. In this paper, we report the most complete transcriptional regulatory network of *M.tb* to date. Capitalizing on previous attempts to build up the *M.tb* transcriptional regulatory network, we have been able to assemble a network that links together the many isolated interactions reported in the literature. Specifically, we cover roughly the 40% of the *M.tb* genome (1624 genes) and report 3212 interactions.

Thinking on a wider perspective than the strictly biomedical interest of this work, our study also address important open questions in the field of network science. During the last decade, it has been shown that many complex systems from fields as diverse as sociology, technology, economy or molecular biology, are made up of components that form interaction patterns or complex networks that share an strikingly rich amount of topological features [11]–[14]. Analyzing the commonalities and differences among the topological features of these heterogeneous networks is of utmost importance. To the best of our knowledge, the network here assembled is the first intracellular pathogen whose TR network is characterized to a reasonable level of accuracy and completeness. In this paper, we calculate the main macro-scale features of the *M.tb* TRN: connectivity distributions and mean values, assortativity and clustering coefficients as well as average path lengths [14]. Of further interest is the analysis of small scale features as given by the abundances and significances of the so-called network motifs, i.e., small subgraphs whose high relative abundances can be identified as strong topological markers. The analysis carried out gives relevant information about the effective tasks for which the network under study is designed or evolved [15], [16]. Finally, we also present a comparative topological analysis between the TR interactome of an intracellular parasite like *M.tb* and other already available analogous system, *E.coli*, with diametrically opposite life styles.

## Results

### Construction of the TR network of *M.tb*

**Figure 1:**
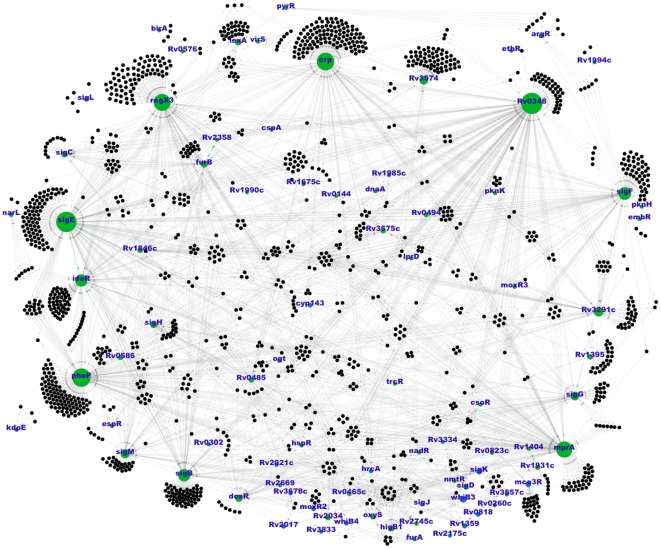
TR Network of *M.tb*. Blue nodes represent regulatory genes that are not regulated by other nodes, while green ones are nodes that regulate the activity of other targets and are regulated by other transcription factors. Self-regulations are represented by black arcs, while feedbacks of mutual regulations are represented in green, thick lines. The picture has been done using the software *Gephi*.

Our starting point is the TR network proposed by Balázsi and colleagues a few years ago [17], which is the largest *M.tb* transcriptional network to date (see Table 1). Based on this TR network, we have performed a considerable expansion by using publicly available sources, most of which appeared after Balazsi's compilation 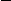
see *Materials and Methods* 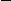. For such an expansion we have used resources that are based on two different experimental groups of methodologies. Within the first family of experimental procedures, we have considered techniques that are based on detecting significant changes of target-gene expression levels caused by disrupting, over-expressing, or inducing a certain regulator, compared with wild type reference expression levels. These techniques include microarrays analysis (genome-wide, poorly specific), or quantitative real time qRT-PCR analysis (that provides higher accuracy and reliability), as well as fusion in target promoters of sequences coding reporters like *gfp* or *lacZ*. On the other hand, the second family of methodologies covers procedures that are based on the identification of the DNA-transcription factor binding sites, and, eventually, the characterization of the physical protein-DNA interaction. Electrophoretic mobility shift assays, one hybrid reporter systems and ChiP-on-chip assays are examples of these methodologies. Moreover, once the new information coming from experimental sources and computational inference is compiled, we have further enlarged the network by operon-based expansion as done in [17], using the operon map predicted in [18]. See *Materials and Methods* for more details. Figure 1 shows the resulting TR network of *M.tb*. We next analyze its main topological properties at different resolution levels.

**Table 1:** Sources considered in [17] to assemble the first TR network of *M.tb*, which constituted our starting point.

Source	Number of genes	Number of links	Number of
			transcription factors
1. Explicit bibliography based network	380	380	26
2. Operon extended version of 1	518	580	26
3. *E.coli* orthologies based network	201	223	29
4. Operon extended version of 3	358	409	29
5. 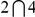	93	53	8
6. Total network ( 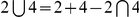 )	783	936	47

The difference of one link and one gene existent between the table and the data provided by Balazsi et al. in [17] is because the link DosR-otsB1 (Rv2006) is counted twice in [17] (See supplementary spreadsheet from [17], lines 484 and 500).

### Global topological properties of the network

**Figure 2:**
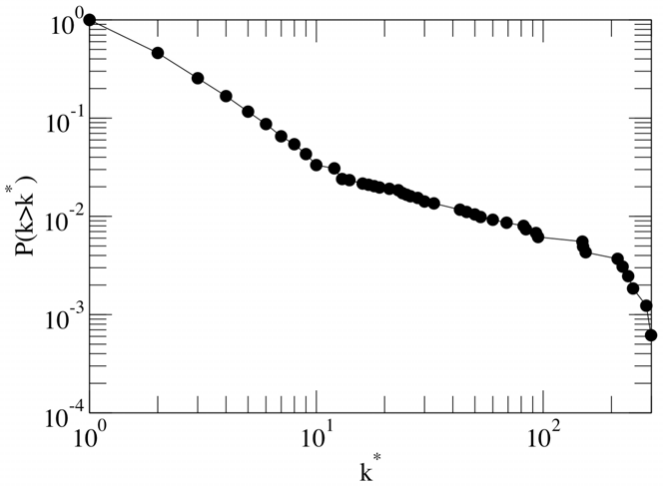
Degree distribution of the TR network of *M.tb*. The figure shows the cumulative degree distribution, i.e., the probability to find a node whose connectivity is larger than or equal to 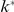. The plot is in log-log scale so that a straight line corresponds to a power law function. The best fit gives an exponent (the slope of the curve) of 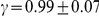.

We have measured several topological properties of the assembled network. It consists of 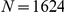 nodes and 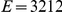 edges, with a giant connected component of order 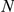. As the network is directed, one can compute the total degree of a node, 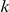, as the sum of the incoming links (meaning that the node is a target of a regulation) and the outgoing links (meaning that the node regulates another node), i.e., 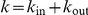. Figure 2 represents the cumulative degree distribution for the TR network of *M.tb*. This distribution gives the probability to find a node with degree 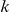 larger than a given degree 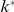. As can be seen from the figure, the cumulative degree distribution follows a power law 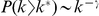, with 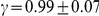. In other words, the TR network of *M.tb* shows the same highly heterogeneous degree distribution found for other biological networks not only at the cellular level, but also at larger scales [14]. This means that the vast majority of genes only interact with a few other genes, while there is a small but statistically significant number of genes that interact with hundreds of genes.

**Figure 3:**
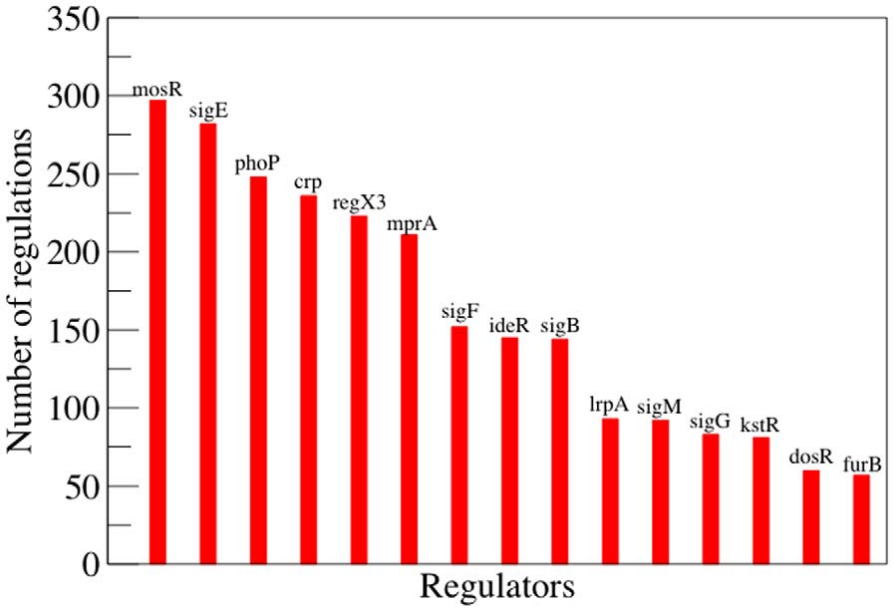
Most connected regulatory hubs in the *M.tb* transcriptional regulatory network. The figure reflects the high heterogeneity of the degree distribution. Namely, there are a few nodes with hundreds of interactions (regulating other genes), while most of the nodes in the network have a few transcriptional relations. For a list of all transcription factors identified see *Materials and Methods*.

Concerning the kind of regulation, a similar plot but only taking into account incoming or outgoing links shows that both in-degree and out-degree distributions are also highly heterogeneous. However, the larger contribution to the many interactions of a few nodes mainly comes from transcription factors (see Figure 3). This can be appreciated already in Table 2, where we have summarized several topological properties. As a matter of fact, the average out-degree of transcription factors is much larger than the average in-degree of genes that have at least one regulator, indicating that most of the hubs are the formers (note that these quantities are not calculated in the usual way, otherwise 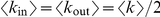). Concerning other topological features, we see that they are within the typical range of values for other biological (and, in general, real complex) networks [14].

**Table 2:** Topological properties of TR network of *M.tb*.

Property	*M.tb* TR network
genes	1624
Transcription factors	83
Links	3212
Self-loops	43
2 nodes feedback loops	6
Mean connectivity	3.96
Mean in-connectivity	2.01
Mean out-connectivity	38.70
Directed average path length (Giant Component)	2.07

We report some global metrics of the network such as its mean connectivity and directed average path length. For the definition of these quantities, see [14]. Note that the mean in and out degrees are calculated with respect to the number of target genes and transcription factors, so they are not normalized in the same way. See the text for more details.

We have also inspected what are the most connected transcription factors, see Figure 3. As one can see, *phoP*, whose inactivation is at the root of the new live vaccine currently being developed, appears as the third most connected hub of the network, only following to *mosR* and *sigmaE* regulators. Indeed, it is known that *PhoP* regulates key functions required for the intracellular survival and persistence of *M.tb*. Admittedly, inactivation of *phoP* results in down-regulation of genes needed to successfully survive within macrophages and consequently in *M.tb* attenuation. Given that the analysis summarized in Figure 3 is straightforward once the network is built up, one can ask whether other transcription factors that share a large number of connections are also key components from a dynamical (functional) point of view, and therefore potential candidates to design new vaccines.

### Small-scale properties of the network: motifs

The previous topological characterization allows to find system wide properties. However, as we shall argue later, mesoscopic or small scale topological features are key to understand the dynamics and function of the networks under study. For instance, communities (subgraphs whose elements are more connected among themselves than with external elements) are often identified with functional modules. Another important tool is the analysis of motifs abundance. A motif is a connected reduced-size subnetwork (typically of length 3 to 5). The statistics of motifs, namely, the number of times that a given motif appears in a network with respect to a certain null ensemble, is a statistically meaningful way of characterizing a network [15], [16]. In order to uncover the structural design principles of complex systems, the study of motif appearances in real networks has emerged as a fundamental tool. As it has been shown [19], different types of natural networks share different profiles of subgraphs significances.

Using an approach similar to that introduced by Alon and coworkers [15], [16] we have firstly registered the number of appearances for each of the subgraphs of length 3 and 4 in the *M.tb* network (see *Materials and Methods*). These numbers, even normalized by the total number of registered graphs, do not tell very much about the relevance of the corresponding motifs since they are strongly biased by the macro-scale features of the network. For example, the fat-tailed connectivity distribution typical of scale-free networks [13] makes single input modules to appear many more times than any other motif. Therefore, to get a better descriptor of motifs significance (i.e., whether or not they are more or less present than usual), we have to compare motifs appearance with a null model, namely with the frequency of motifs that comes out in an ensemble of suitably randomized networks. We have used the approach firstly suggested in [20]. It consists of generating, from the initial system, networks that preserve the same connectivity sequence of the original one. To this end, we implement a switching algorithm that preserves not only the number of incoming and outgoing links of each node, but also the number of mutual links when this is the case in the original TR network (see *Materials and Methods*). This kind of randomizing procedure has been the subject of intense research in the last years, and besides the method used in this paper, there are other alternative randomization schemes [16], [20], [21].

Once the ensemble of randomized networks is generated, we calculate the mean values and the typical deviations of the number of appearances of each of the possible motifs of a given length in all the randomized networks. The significance of each motif 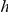 is determined by the so-called Z-Score as follows,
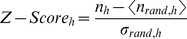
(1)


At this point, it is worth noticing that when a subgraph is absent in any of the randomized networks of the null model, the Z-Score for that precise subgraph cannot be defined. Moreover, all the defined Z-scores are normalized considering each of them to be a component of a unitary vector. This normalization allows to compare motifs significance profiles of networks of very different sizes.

**Figure 4:**
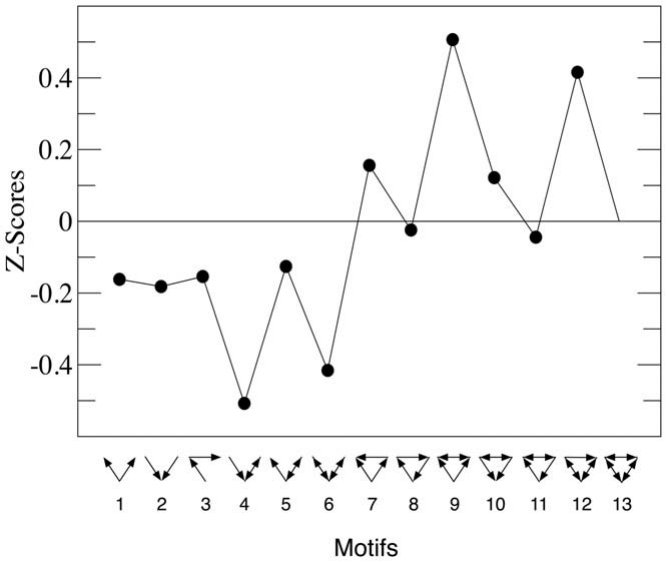
Triads significance profile of the *M.tb* TR network. We represent the values of the Z-scores as defined in Eq. (1) for each of the 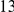 possible 3-nodes directed motifs, which are depicted in the x-axis. Only one of them cannot be defined in the *M.tb* network. See the main text for further details as well as the section *Materials and Methods*.

We have calculated the significance profiles for the newly assembled TR network of *M.tb* focusing on 3 and 4 nodes' motifs. Figure 4 shows the Z-scores obtained for triads. Note that as our network is directed, so are the triads. Out of 13 possible motifs of length 3, only triad 13 is not present in the Z-scores representation. Additionally, the first six motifs, all of which correspond to open structures (i.e., loopless motifs) are underrepresented in the *M.tb* transcriptional network, while those that are found more frequently than in the random version of the TR network are closed motifs.

## Discussion

The statistics of motifs as represented in Figure 4 has been used as a way to deepen our understanding of the relation between the structure and function of biological networks. Specifically, previous works have focussed on directed triads as a means to categorize networks around superfamilies which, roughly, share the same functionality even when the networks belong to very different fields [19]. Two distinct superfamilies were identified for informational-processing networks [19]. In one of these superfamilies, we can find networks for which the response of the whole system to an stimulus cannot last much more than the response time of one of its interactions. These so-called *rate-limited* networks include TR networks of unicellular microorganisms, covering both prokaryotic and eukaryotic organisms. The second informational processing superfamily groups networks that work in a less immediate way, being able to perform slower responses with characteristic times that can be even several orders of magnitude greater than the response times of its single interactions. Synaptic and developmental networks in multicellular organisms are typical cases of this kind of *unrate-limited* behaviors.

The question is then how the TR network of an intracellular parasite like the *M.tb* integrates into the above framework, i.e., which of the two informational processing superfamilies the *M.tb* TR network belongs to. It is worth mentioning that *a priori* the question is not trivial. All unicellular microorganisms have presented up to now the characteristic profiles of rate-limited networks [19], so that one may be tempted to ascribe to this family the network of *M.tb*. However, the bacillus spent most of its vital cycle within the macrophage, so that the characteristic response times of its TR network should be slower. Several findings support this hypothesis. For instance, it has been reported [17] that after hypoxia stimulus, the dynamics stabilizes in a time as long as eighty days, suggesting that the TR network of *M.tb* could better adjust to the unrate-limited superfamily. As a matter of fact, a direct comparison of the triads profile of *M.tb* with those reported in [19] shows that this is indeed the case.

Beyond the question of network superfamilies, it is also of interest to compare the TR networks of *E.coli* and *M.tb* as two examples of prokaryotic, unicellular bacteria. The aim is to identify whether there are relevant divergences in their motifs profiles that could be related to their different vital cycles: that of an intracellular parasite in the case of *M.tb* and that of an extracellular bacterium in the case of *E.coli*. Special care have to be taken with motifs containing feedback loops. Feedback loops are considered as rare structures in TR networks of unicellular microorganisms because of their scarce presence in most of the best characterized TR networks [19]. For instance, none of them were present in the original TR network of *E.coli*, and only one in that of the Yeast [19]. However, with the proliferation of new experimental data in the last years, we can find as many as 10 feedback loops in the updated network of *E.coli* (see *Materials and Methods*) and 5 in the *M.tb* network. These numbers, even being small with respect to the total size of the systems, give high motifs significances in some of the subgraphs where they are present.

**Figure 5:**
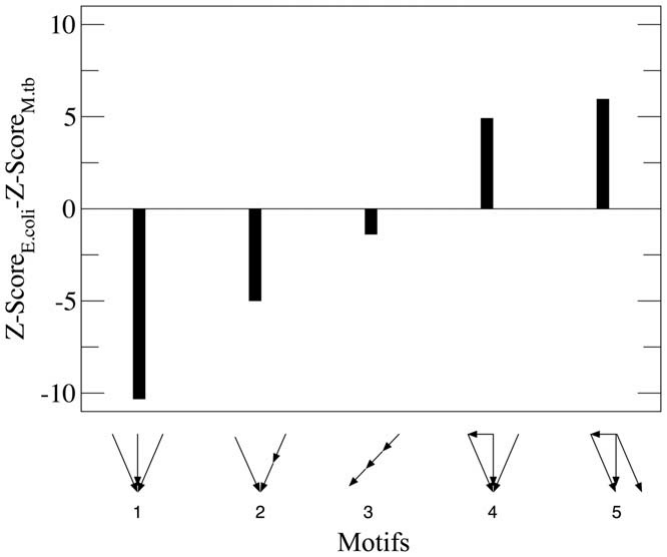
Differentially significant motifs present in *M.tb* and *E.coli* TR networks. Feedback-free tetrads present more than 1000 times in both networks were divided into three groups according to their Z-scores: we consider as overrepresented those tetrads with Z-Score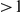 and as underrepresented those with Z-Score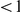, being the third group joined by tetrads for which 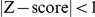. We have only looked for motifs that belong to different groups in *E.coli* and in *M.tb*, and we have sorted them according to the absolute difference 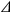
Z
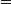
Z-Score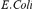-Z-Score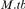. After this filtering process, the tetrads in the figure are those with 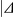
Z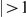. The resulting tetrads with highest 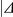
Z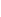 are the same when one takes the normalized Z-score.

Taking into account the above remarks, we have compared the Z-scores significance profiles for the networks of *E.coli* and *M.tb* looking for common (i.e., present in both organisms) structures that exhibit the larger differences in their Z-scores. To this end, the statistical analysis of tetrads is more convenient. In our analysis, we only consider feedback-free tetrads that appear in both TR networks at least a thousand times. Within this subset, we have selected the structures with a differential behavior in both systems. The results are depicted in Figure 5, where we have represented the cases for which the differences in Z-scores between tetrads of both networks are greater than unity. As one can see in the figure, *E.coli* presents stronger trends for the two simplest parallel combinations of feedforward loops with single regulations (tetrads 4 and 5). On the other hand, the three structures with highest differences in favor of *M.tb* are single output modules (tetrad 1), cascades, (tetrad 3) and the combination of them (tetrad 2). The preference for different motifs in *E.coli* and *M.tb* TR networks supports the evolutionary pressure hypothesis put forward in [15], [16], [19]. At a glance, given the homology between the two networks 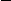 both organisms are bacteria 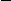, one might think that their TR networks come from common underlying mechanisms that regulate the way they grow and are assembled. However, the TR networks should have been shaped in relation to the vital needs of both bacteria, which are known to be radically different. Therefore, over and under represented subgraphs with different significances in these two organisms are a consequence of the different life cycles and of an evolutionary origin.

From a dynamical point of view, the previous results also make sense. The dynamics associated to most of the motifs that appear to be under or over represented in the TR networks of *M.tb* or *E.coli* have been well characterized previously [15], [22]-[28]. For instance, feedforward loops have been described as a discriminator between persistent and transient signals [15], [22] or as a pulse-generator to accelerate signal responses [23]. The performance of the motif as persistence discriminator or accelerator depends, essentially, on the signs of the regulations and the logical scheme[29], but nevertheless, both functions are useful for the organism when it is subjected to a highly dynamic or unpredictable environment. This is the case of *E.coli* whose environment offers a very rich amount of nutrients to the bacterium, but also a considerable concentration of harmful substances and other threats. This highly variable environment (due to thermic, chemical or Ph-related changes) requires a valuable mechanism to filter the very noisy signals that are being sensed continuously. Therefore, it is to be expected that feedforward and closely related structures be over represented in the *E.coli* TR network.

On the other hand, as noted before, the *M.tb* bacillus expends, on average, more than 90% of its vital cycle sporulated, surviving under a latency regime within the cytoplasm of the human immune system macrophages. In this sense, it is difficult to conceive a bacterial environment more hostile than that of *M.tb*. However, hostility does not mean variability, so that the environment of *M.tb* could be hardly less volatile or more predictable than the macrophage cytoplasm. These considerations are in qualitative agreement with our observations of a less marked bent for feedforward loops in *M.tb*.

### Conclusion

In this work, we have assembled the more complete TR network of *M.tb*. to date. The network has been built up by exhaustively including publicly available bibliographical information relative to microarray essays, protein-promoter binding sites determination experiments and synthetic biology techniques coming from as many as 31 different works. The importance of gathering all this information into a common frame with tractable format is twofold. First, it is a valuable database that can be directly used for research purposes. And second, the new network constitutes an important tool for the application and development of computationally inspired models and methods that may be able to guide future in-vitro and in-vivo experiments. The latter includes using the network to develop new tools for tasks such as the identification of spurious links and missing interactions [30], prediction of unknown functions of the proteins, the generation of more accurate operon maps predictions [18] or the dynamical modeling of network operations as a sensory system [31]. Concerning the role of *phoP*, our analysis shows that it appears as the third-most connected hub of the network (see Figure 1), thus, the relevance of altering its functionality is easily understandable and expected from a network viewpoint. The extended TR network presented in this work is essentially the backbone of the regulatory system, and our topological analysis also reveals what experimental works are demonstrating to be relevant in relation to *phoPR* [6], [32]. This is the reason why we believe that the newly assembled network can provide valuable hints of potential targets (mainly those that, as *phoP*, are the hubs of the network).

Additionally, we have performed a detailed analysis of the topology of the TR network. The results show that our system shares the main macro-scale features of TR networks such as the small-world property, assortativity or a fat-tailed degree distribution [13]. The statistical characterization of the relative abundance of different motifs has also shown interesting results. First, our new system could reasonably be incorporated into the differentiation scheme firstly proposed in [19]. Secondly, we have performed a comparative analysis between the significance levels of the most relevant tetrads in the TR networks of *E.coli* and *M.tb*. Our results show that the relative abundance of the different subgraphs is tightly related to the dynamical response of each subgraph [15], [22]–[28] and to the life styles (in relation to their environments) of both bacteria. Besides, given that the TR networks correspond to two bacteria, the differences between subgraph significances of *E.coli* and *M.tb* can only be a consequence of divergent evolutive pathways.

In summary, the expanded TR network will be useful to provide an overview of multiple functional aspects of *M.tb*, and to suggest new experiments. It is also important to note (especially for future studies) that the network obtained does not distinguish between activation and inhibition in gene expression, which should be introduced in order to develop considerations of broader functional scope. The same is true with regard to the relative quantification of changes in the expression (introduction of “weights” on the links between nodes). Besides, the methodology used to expand the network and the four kinds of resources used, which have been treated case by case, individually for each transcription factor, allow for a quick and easy review and extension of the network. Finally, the development of a functional sense upon the extended TR network of *M.tb* that encompasses the whole TR network and the integrative action of the signaling system, would be one of the essential objectives to achieve in the near future.

## Materials and Methods

### Bibliographical revision and datasets of the TR network of *M.tb*

We have updated the network presented in [17], using new and copious experimental information available from as many as 31 different works dated in the last ten years, (see Table S1). To assemble and expand the network, we have also used the predicted operon map proposed in [18] assuming that if a transcription factor A regulates a gene B belonging to an operon BCD, then, also the interactions A–C and A–D are present.

All the information extracted is given at Table S2, including the experimental works where the regulations contained in Balázsi's compilation were originally reported. Finally, we filtered all repeated information to build our network. The final system, as can be seen in Table 2, contains 1624 nodes (genes) and 3212 links, i.e., more than two times the size of the previously available dataset [17]. The expanded network can be found in Material S1, in.txt format.

### *E.coli* TR network

We have used the TR network of *E.coli* [33] updated as of August 2010 (release 6.8), which contains experimental information until the ultimate large-scale revision published in 2008 [34]. Dimeric transcription factors and toxin/antitoxin systems are taken as single nodes of the network. The network can be found in Material S2, in txt format.

### Subgraph counting algorithms

In order to evaluate the motifs significance profiles, we have developed algorithms that randomize the original network and count triads and tetrads in both the original network and the set of randomized systems. All algorithms have been programmed in C and are provided in three independent files. Each one of them performs the following tasks:

• randomizer.c (Material S3): it takes the original network and generates a set of randomized versions of it. The size of the network, 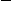
number of nodes and links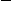 is also read by the algorithm from the file “Parameters.txt”, as well as the desired number of random networks to create. So, this file “Parameters.txt” must contain only these three numbers separated by blank spaces. The original network should be provided in a two column file “Network.txt”. The program follows the scheme proposed in [19]. Essentially, we randomize separately mutual links and simple links, avoiding interferences between these two kinds of two-nodes structures (mutual links rewire with mutual links and simple links rewire with simple links). So, for each random network, we start by choosing at random a number of rewirings to be performed for the simple links (between 100 and 200 times the total number of simple links in the network). Then, for each potential rewiring we choose two single links at random, and exchange them provided that they do not generate self-loops, mutual links or multiple links (see Figure S1, panel a). Otherwise the rewiring is not accepted (nevertheless it is counted). Rewiring of mutual links proceeds in a similar way, always taking care of potential conflicts (see Figure S1, panel b). The output of this algorithm will be a file called “Networks.txt”, which stores the original network together with the ensemble of randomized versions, one after another, always in a two column format of the form regulator-target. All the results presented in this work have been obtained by taking 100 random networks.

• triads.c (Material S4): the algorithm takes the output file that generates randomizer.c, and identifies and counts the triads present in both the original network and any of the random ones. Essentially, the algorithm:

1. needs to read the number of nodes and links of the original system and the number of random networks from “parameters.txt”, as well as the list of links of all the systems in “Networks.txt”.
2. registers the nodes belonging to each triad and write them in the three column file “TriadsCensus.txt”, in which we can find the three nodes of each triad (different triads correspond to different rows). This file lists the triads that belong to all the networks, one after another. For this reason, the algorithm also saves the total amount of triads in each of the networks (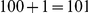 in our case) and stores them in “TriadsPerNetwork.txt”, in order to identify the lines of “TriadCensus.txt” that are referring to each network.
3. identifies the type of each registered triad for each network, by checking its “topological footprint” and comparing it with the list of all possible types [35] in “triadsIDs.txt” (available at Material S6). The program counts the total amount of triads of each type present in each network and register them in “TriadsCounts.txt”.

• tetrads.c (Material S5): The strategy performed by this algorithm to exhaustively count all tetrads as fast as possible starts by reading the file “TriadsCensus.txt” created by triads.c. Next, systematical sweeps of the fourth gene are performed taking care of not registering tetrads more than once. Specifically:

1. the code registers the number of tetrads of each type existent in each network. To do this, the number of nodes, links and random networks are read from the file “Parameters.txt” and the triads are taken from “TriadCensus.txt” to perform the sweeps on the fourth gene. Besides, in order to know what triads belong to each of the 101 networks -in our case- sequentially analyzed, the code reads “TriadsPerNetwork.txt”.
2. for each identified tetrad, we check its “topological footprint” and determine its type by comparing it with all the 199 possible types listed in “tetradsID.txt”(Material S7). It is worth mentioning that while only six numbers are enough to unambiguously identify a certain triad (the in and out connectivities of each of its three nodes), we have needed thirteen numbers as topological coordinates to correctly distinguish between the 199 kinds of tetrads. Using these topological footprints, we reproduce the scheme of the “motifs dictionary” available in [35].
3. the code counts the total amount of tetrads of each type present in each network and register them in “TetradsCounts.txt”.

The informational flow between executable files and the.txt files created and/or read by the algorithms is illustrated in Figure S2. With the information coming out from these files, the Z-scores corresponding to triads and tetrads can be straightforwardly calculated.

## Supporting Information

Figure S1 The figure represents the set of allowed and forbidden rewiring steps for the randomization of the TR network. The left panel corresponds to the situation in which simple links are being rewired whereas the right panel represents the cases considered when mutual links are being rewired.
(TIFF)

Figure S2 Flow between the different codes and files used to determine the Z-scores of triads and tetrads. The source of the codes used are provided as Supplementary Material. The files “network.txt” and “Parameters.txt” are not explicitly provided.
(TIFF)

Material S1 Transcriptional Regulatory network of *Mycobacterium tuberculosis* dataset.
(TXT)

Material S2 Transcriptional regulatory network of *Escherichia coli* dataset.
(TXT)

Material S3 Randomizer.c: code used to generate a null ensemble of random networks.
(C) Click here for additional data file.

Material S4 Triads.c: code used to identify triads.
(C) Click here for additional data file.

Material S5 Tetrads.c code used to identify tetrads.
(C) Click here for additional data file.

Material S6 Triads.txt defines the topological fingerprint of each of the 13 possible triads.
(TXT)

Material S7 Tetrads.txt defines the topological fingerprint of each of the 199 possible tetrads.
(TXT)

Table S1The Table contains the references used to build up the TR network reported in the main text as well as the transcription factors studied. 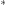, 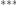 and 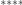: These works were already cited in [17], nevertheless, not all the regulations reported in these works were considered in the compilation of Balazsi et al. More precisely, we have found 18 regulations of DosR coming from Park at al and 110 links coming from Manganelli et al not included in the previous database. 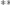 reports regulations coming from the following 31 transcription factors: *oxyS, Rv0260c, sigK, regX3, Rv0818, Rv0823c, mprA, sigE, Rv1359, Rv1931c, higB1, Rv1990c, Rv2017, Rv2021c, Rv2034, Rv2175c, Rv2669, sigB, Rv2745c, dosR, moxR3, sigJ, Rv3334, sigD, whiB3, Rv3557c, Rv3678c, whiB4, moxR2, nmtR, Rv3833*.(PDF)

Table S2 Exhaustive compilation of all the information regarding the bibliographical revision process: links, works in which they had been reported and experimental methodologies used to infer them.
(XLS)
