# Supplementary figures and images for: The Transcriptional Regulatory Network of Mycobacterium tuberculosis
Source: PLoS One. 2011 Jul 19;6(7):e22178. doi: 10.1371/journal.pone.0022178 (PMC3139605; doi:10.1371/journal.pone.0022178)

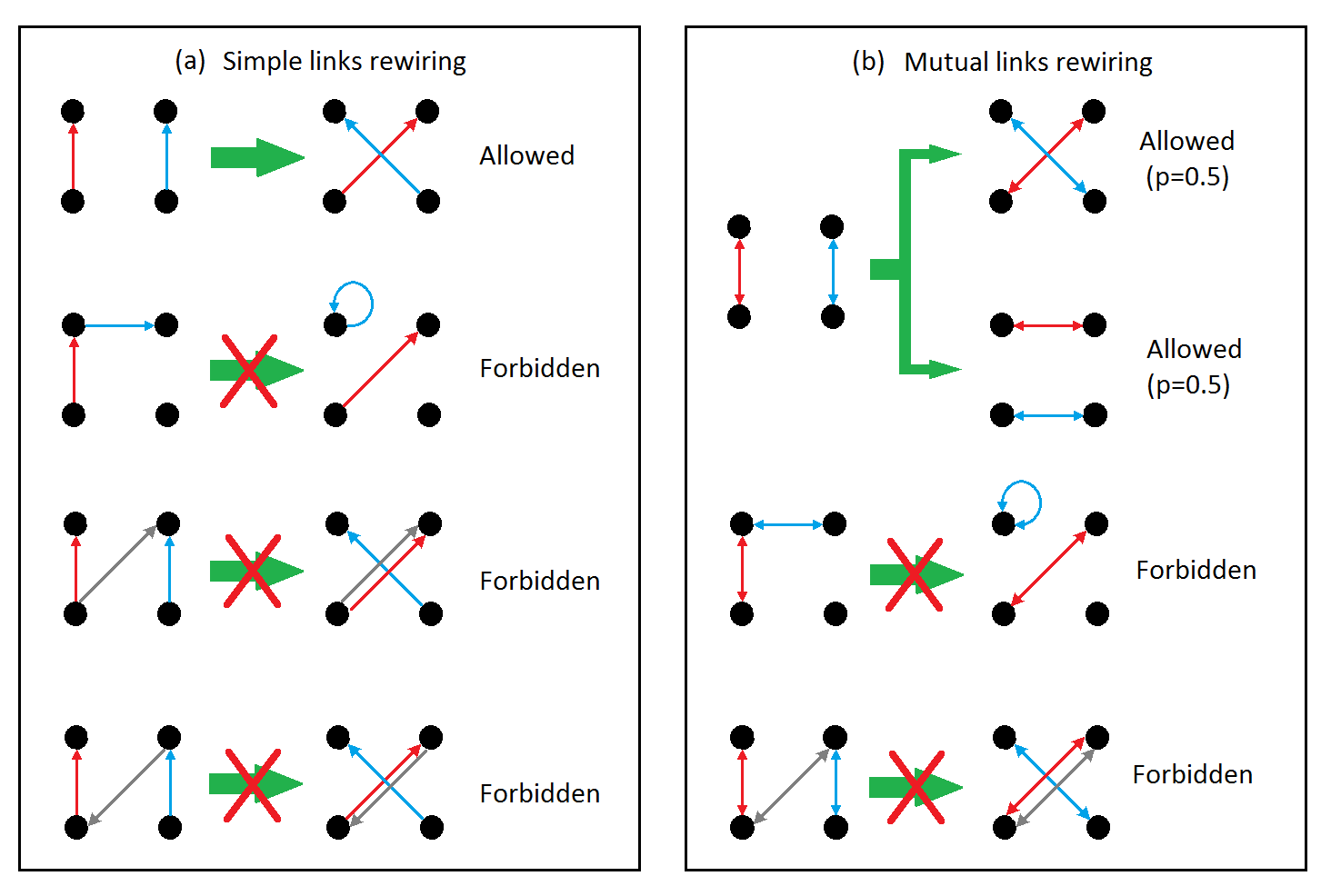

Supplement: Figure S1 — The figure represents the set of allowed and forbidden rewiring steps for the randomization of the TR network. The left panel corresponds to the situation in which simple links are being rewired whereas the right panel represents the cases considered when mutual links are being rewired. (TIFF) [file pone.0022178.s001.tif]

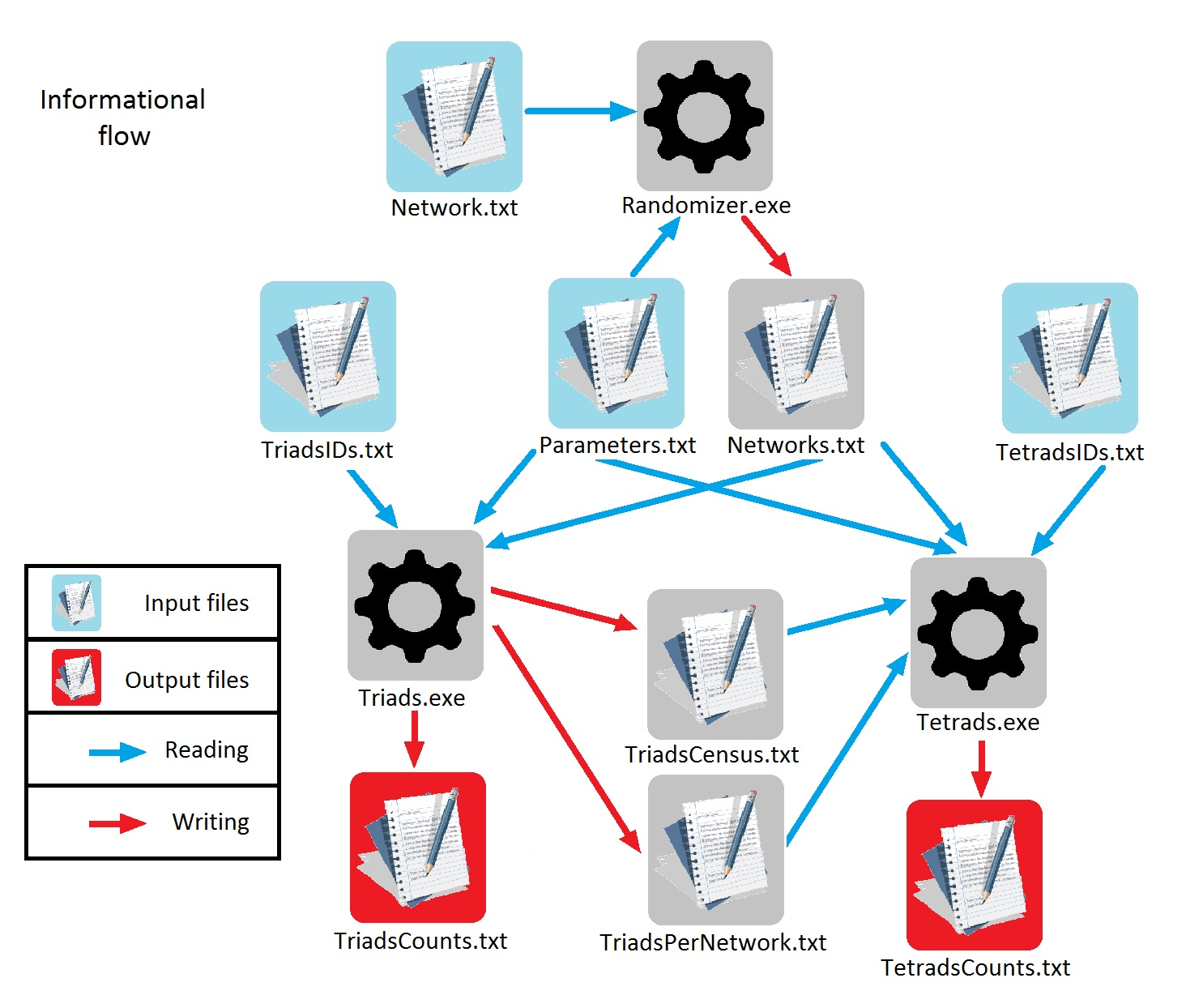

Supplement: Figure S2 — Flow between the different codes and files used to determine the Z-scores of triads and tetrads. The source of the codes used are provided as Supplementary Material. The files “network.txt” and “Parameters.txt” are not explicitly provided. (TIFF) [file pone.0022178.s002.tiff]
