# Supplementary material for: The Transcriptional Regulatory Network of Mycobacterium tuberculosis
Source: PLoS One. 2011 Jul 19;6(7):e22178. doi: 10.1371/journal.pone.0022178 (PMC3139605; doi:10.1371/journal.pone.0022178)
Supplement: Table S1 — The Table contains the references used to build up the TR network reported in the main text as well as the transcription factors studied. , and : These works were already cited in [17], nevertheless, not all the regulations reported in these works were considered in the compilation of Balazsi et al. More precisely, we have found 18 regulations of DosR coming from Park at al and 110 links coming from Manganelli et al not included in the previous database. reports regulations coming from the following 31 transcription factors: oxyS, Rv0260c, sigK, regX3, Rv0818, Rv0823c, mprA, sigE, Rv1359, Rv1931c, higB1, Rv1990c, Rv2017, Rv2021c, Rv2034, Rv2175c, Rv2669, sigB, Rv2745c, dosR, moxR3, sigJ, Rv3334, sigD, whiB3, Rv3557c, Rv3678c, whiB4, moxR2, nmtR, Rv3833. (PDF) [file pone.0022178.s010.pdf]

Table S1: Experimental sources for the M.tb TR network.

Reference	Strain	Methodologies	Regulator(s)	Links
1. Gonzalo-Asensio, J. et al., *J. Biol. Chem.*, **281**, 1313 (2006).	MT103	Microarray	*phoP*	78
2. Walters, S.B. et al., *Mol. Microbiol.*, **60**, 312 (2006).	H37Rv	Microarray, qRT-PCR	*phoP*	114
3. Parish, T. et al., *Microbiology*, **149**, 1423 (2003).	H37Rv	Microarrays	*RegX3*	98
4. Park, H.D. et al., *Mol. Microbiol.*, **48**, 833 (2003).	H37Rv	Microarray, EMSA, identification of consensus sequences (CS)	*dosR*	49*
5. Santangelo, M. et al., *Tuberculosis*, **89**, 22 (2009).	H37Rv	LacZ-promoter fusion,EMSA	*mce2R*	14
6. Abomoelak, B. et al., *J. Bacteriol.*, **191**, 5941 (2009).	H37Rv	Microarray, qRT-PCR, EMSA	*mosR*	173
7. Maciag, A. et al., *J. Bacteriol.*, **189**, 730 (2007).	H37Rv	Microarray, qRT-PCR, CS, EMSA	*FurB*	32
8. Guo, M. et al., *Genome Res.*, **19**, 1301 (2009).	H37Rv	one hybrid reporter system	**	325
9. He, H. et al., *J. Bacteriol.*, **188**, 2134 (2006).	H37Rv	Microarray, qRT-PCR, EMSA	*MprA*	135
10. Fontan, P. et al., *J. Infect. Dis.*, **198**, 877 (2008).	H37Rv	Microarray, qRT-PCR	*sigE*	16
11. Manganelli et al., *Mol. Microbiol.*, **41**, 423 (2001).	H37Rv	Microarray, CS	*sigE*	116***
12. Williams, E.P. et al., *J. Bacteriol.*, **189**, 4234 (2007).	CD1551	Microarray, qRT-PCR, CS, In vitro transc. assays (IVTA)	*sigF*	74
13. Raman, S. et al., *J. Bacteriol.*, **188**, 8460 (2006).	H37Rv	Microarray, CS	*sigM*	43
14. Lee, et al. *J. Bacteriol.*, **190**, 1128 (2008).	CD1551	Microarray, qRT-PCR, CS, IVTA	*sigG*	43
15. Lee, et al. *J. Bacteriol.*, **190**, 699 (2008).	CD1551	Microarrays, qRT-PCR, CS, IVTA	*sigB*, *sigF*	78
16. Akhter, Y. et al., *Gene*, **407**, 148 (2008).	H37Rv	CS	*crp*	44
17. Micklinghoff, J.C., et al., *J. Bacteriol.*, **191**, 7260 (2009).	H37Rv	Microarray, qRT-PCR, EMSA	*RamB*	4
18. Raghavan, S., et al., *Nature*, **454**, 717 (2008).	Erdman	Microarray, qRT-PCR, EMSA	*EspR*	12
19. Reddy, M.C.M. et al., *Prot. Sci.*, **17**, 159 (2008).	H37Rv	EMSA, CS	*LrpA*	15
20. Gazdik, M.A. et al., *Mol. Microbiol.*, **71**, 434 (2009).	BCG,H37Rv	Microarray, qRT-PCR, proteomic studies, EMSA	*cmr*	5
21. Agarwal, et al., *Infect. Inmun.*, **75**, 452 (2007).	CD1551	Microarray, qRT-PCR, CS	*sigM*	25
22. Andreu Martín, N., *Doctoral Thesis*,	H37Rv	Microarray	*Rv0576*	2
23. Rodriguez, G.M. et al., *Infect. Inmun.*, **70**, 3371 (2002).	H37Rv	Microarray	*IdeR*	71
24. Liu, T. et al., *Nat. Chem. Biol.*, **3**, 150 (2006).	H37Rv	qRT-PCR, EMSA	*CsoR*	4****
25. Kantardjieff , K.A., et al., *Acta Cryst.*, **D61**, 355 (2005).	H37Rv	Protein structure based inferences	*pyrR*	6
26. He, H. & Zahrt, T.C. *J. Bacteriol.*, **187**, 202 (2005).	H37Rv	qRT-PCR, EMSA, CS	*mprA*	3
27. Singh, A. et al. *FEMS Microbiol. Lett.*, **227**, 53 (2003).	Erdman	LacZ-promoter fusion, gfp-promoter fusion	*VirS*	8
28. Santangelo, M. et al., *Microbiology*, **155**, 2245 (2009).	H37Rv	Microarray, qRT-PCR, Proteomic studies, CS	*Mce3R*	27
29. Kendall, S.L. et al., *Mol. Microbiol.*, **65**, 684 (2007).	H37Rv	Orthology (*M.smegmatis*), EMSA, proteomic studies, CS	*KstR*	77
30. Goldstone, R.M. et al., *Infect. Inmun.*, **77**, 4654 (2009).	H37Rv	Microarray, qRT-PCR	*Rv0485*	13
31. Sala, C. et al., *Mol. Microbiol.*, **71**, 1102 (2009).	H37Rv	qRT-PCR, EMSA, ChiP-on-chip, CS	*BlaI*	16
